# Complete genome sequence of the *Anaplasma phagocytophilum* clinical isolate NCH-1

**DOI:** 10.1128/mra.00706-26

**Published:** 2026-09-08

**Authors:** Travis J. Chiarelli, Daniel P. Miller, Jason A. Carlyon

**Affiliations:** 1 Department of Microbiology and Immunology, School of Medicine, Virginia Commonwealth University Medical Center 72054, Richmond, Virginia, USA; Queens College Department of Biology, Queens, New York, USA

**Keywords:** *Anaplasma phagocytophilum*, long-read, obligate intracellular bacterium, Nanopore, whole-genome sequence, bacterial isolation

## Abstract

*Anaplasma phagocytophilum* is an obligate intracellular gram-negative bacterium and etiologic agent of human granulocytic anaplasmosis. *A. phagocytophilum* genomic sequencing has historically been performed via short-read platforms. Our optimized bacterial isolation protocol combined with Nanopore sequencing produced a single, closed 1,481,805 bp circular *A. phagocytophilum* strain NCH-1 chromosome.

## ANNOUNCEMENT

*Anaplasma phagocytophilum*, the etiological agent of human granulocytic anaplasmosis, is a tick-borne pathogen and the most-reported rickettsial disease in the United States (1–4). NCH-1, isolated from a patient at Nantucket Cottage Hospital, Massachusetts, in 1994, is a widely used laboratory reference strain (5). *A. phagocytophilum* genomes are highly repetitive (~13%), with *msp2/p44* homologs comprising ~5% alone (6). Host DNA contamination due to *A. phagocytophilum’s* intracellular lifestyle has historically limited genome sequencing efforts to short-read technologies. This, combined with the genome’s repetitive structure, raises concerns about assembly completeness and accuracy (7). By developing an improved bacterial isolation methodology and employing Oxford Nanopore Technologies (ONT) long-read sequencing, we produced a complete *A. phagocytophilum* strain NCH-1 genome assembly.

**Fig 1:**
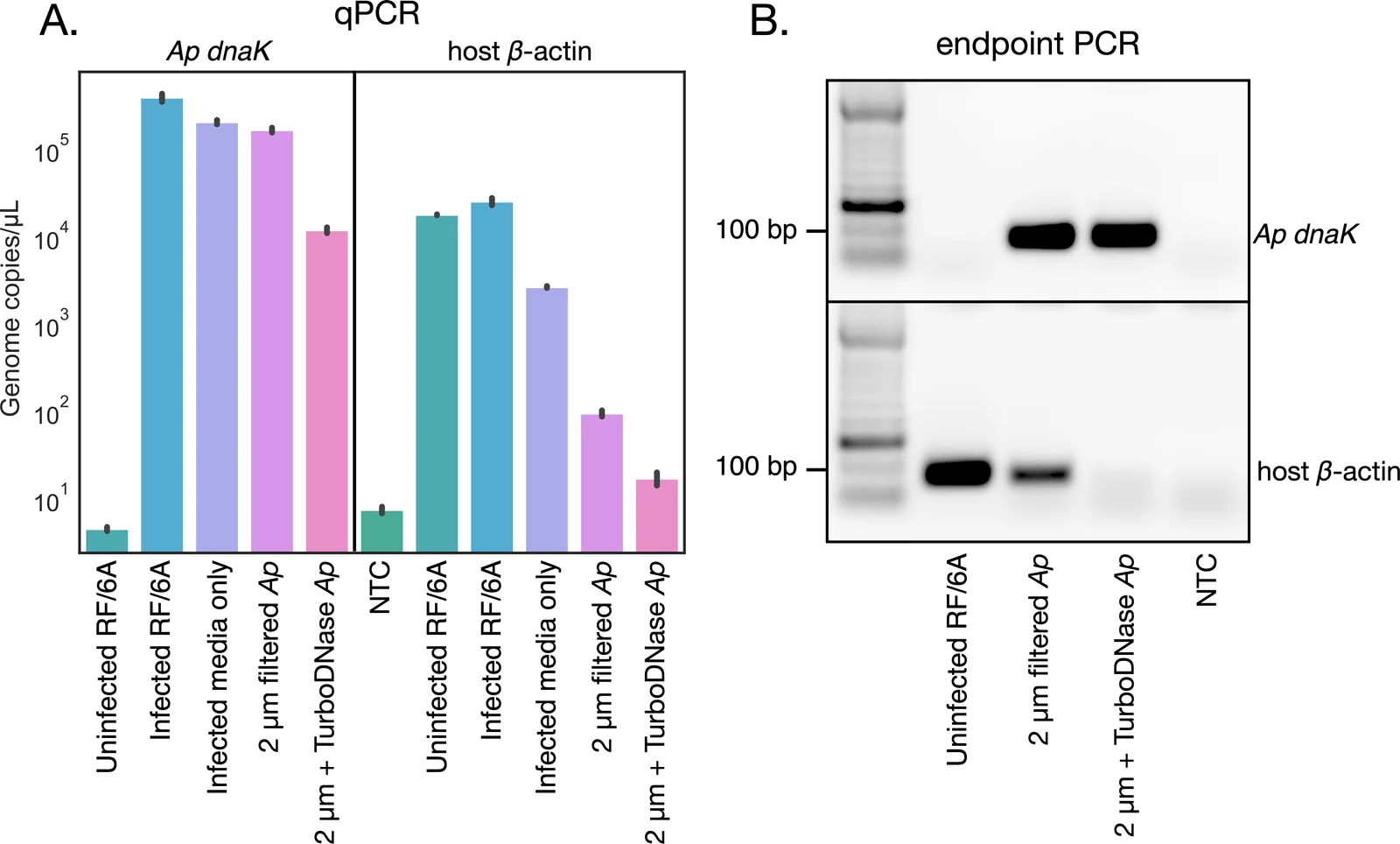
*A. phagocytophilum* genome isolation for subsequent ONT sequencing. (**A and B**) q- and endpoint-PCR assessing *A. phagocytophilum* (*Ap*) DNA purity with primers against *Ap* strain NCH-1 *dnaK* (5′-atggcggctgagcgtataatagg-3′, 5′-ctaagtattcttcttgtcctcgtccttattaatctcc-3′) and host β-actin (5′–agatgacccagatcatgtttgagacct–3′, 5′–tcgtagatgggcacagtgtgg–3′) genes. Prior to *A. phagocytophilum* genomic extraction, bacterial isolation occurred in three steps: filtering naturally released bacteria through a 2 µm filter, treating the intact bacteria with TURBO DNase and RNase A to digest host-contaminating DNA, followed by *A. phagocytophilum* density pad (30% MD-76R) purification. Multiple samples were taken throughout the process to assess the purity of the *A. phagocytophilum* genomic DNA. After successful *A. phagocytophilum* genomic isolation, samples were sent for whole-genome sequencing.

*A. phagocytophilum* was maintained in RF/6A choroidal endothelial cells (ATCC CRL-1780) (8). Bacteria were isolated from synchronously infected cells at 26 h post-infection (8). Culture media containing naturally released bacteria were collected, filtered through a 2-µm membrane, and centrifuged at 20,000 × *g* for 20 min, 4°C. Pelleted bacteria were resuspended in 1× Hanks balanced salt solution (HBSS: [Gibco]) with brief sonication (10 s burst at 30 amps) using a S-4000 Ultrasonic Liquid Processor (Misonix) to rupture host nuclei. Host nucleic acids were removed by treatment with 1× TURBO DNase buffer (Invitrogen), 6U TURBO DNase (Invitrogen), and 50 µg/mL RNase A (Invitrogen) in 500 µL total volume, followed by purification over a 30% MD-76R density pad via centrifugation at 21,100 × g for 45 min, 4°C (9). Genomic DNA was extracted using the Wizard Genomic DNA Purification Kit (Promega). Preceding lysis, bacteria were resuspended in 100 µL 1× phosphate-buffered saline with 6 mAU Proteinase K (Invitrogen) and incubated for 1 h, 60°C. Host DNA contamination was assessed by q- and endpoint-PCR (8) (Fig. 1). DNA (50 ng/µL; 50 µL) was submitted to Eurofins Genomics for Nanopore sequencing using their Bacterial Genome Sequencing Analysis Pipeline v1.0. Libraries were prepared from unsheared, non-size-selected DNA using the V14 Ligation Sequencing Kit (ONT: SQK-LSK114) and sequenced on a GridION R10.4.1 flow cell.

Default parameters were used for sequence analysis. Basecalling was performed using MinKNOW-v25.05.12 with Dorado. Sequencing generated 168,303 raw reads, totaling 631,905,285 bp. FastQC-v0.12.1 determined a median Phred quality of 31 (10). Quality filtering of 1,000 bp minimum read length was performed with Filtlong, followed by Rasusa downsampling to 180× target coverage (11). Assembly was performed with Flye-v2.9.6-b1802 and polished with Medaka-v2.2.0 (12). Sequences assembled into a 1,481,805 bp contig (QUAST N_50_: 1,481,805; L_50_: 1; L_90_: 1), with 41.65% GC content. CheckM-v1.0.13 with database 2015_01_16 predicted genome completeness at 99.57%, 5.21% contamination (13). Circularization was confirmed with Geneious (Dotmatics) *de novo* assembler, achieving coverages ranging from 271× to 516× with no structural gaps. PGAP-v6.11 predicted 1,163 total, 1,012 protein-coding, 3 rRNA, 37 tRNA, 4 ncRNA, and 107 pseudogenes (14). When compared with alternative isolate assemblies, NCH-1 (GCF_000964725.1), HZ (GCF_000013125.1), and HGE1 (GCF_000478425.1), OrthoANIu (CJ Bioscience) calculated average nucleotide identities of 99.93%, 99.92%, and 99.69%, respectively (15), with most alignment differences localized to contig boundaries and *msp2/p44* loci, strongly suggesting errors in sequence assembly efficacy or differences in genome makeup.

## Data Availability

This project has been deposited at DDBJ/ENA/GenBank under the accession JBZEGS010000000 and the Sequence Read Archive (SRA) accession SRP711862 under BioProject PRJNA1476208, BioSample SAMN60691665.
